# Safety and immunogenicity of an adjuvant-free peptide vaccine targeting IL-17A: Phase 1 randomized controlled trial

**DOI:** 10.1016/j.isci.2026.116817

**Published:** 2026-07-21

**Authors:** Satoru Kasahara, Akiko Tenma, Makoto Sakaguchi, Shigeyoshi Tsuji, Hideki Tomioka, Ryuichi Morishita, Hironori Nakagami, Tetsuya Tomita

**Affiliations:** 1 R&D, FunPep Co., Ltd., Osaka, Japan; 2 Department of Rehabilitation, Orthopaedic Surgery, Psoriasis Center, Nippon Life Hospital, Osaka, Japan; 3 Department of Clinical Gene Therapy, The University of Osaka Graduate School of Medicine, Osaka, Japan; 4 Department of Health Development and Medicine, The University of Osaka Graduate School of Medicine, Osaka, Japan; 5 Graduate School of Health Science, Morinomiya University of Medical Sciences, Osaka, Japan

**Keywords:** epitope, IL-17A, peptide vaccine

## Abstract

We investigated whether a peptide vaccine (FPP003) composed of B cell epitope for IL-17A and T cell epitope (AJP001) with innate immune activation could induce the antibody production without adjuvants. The safety and immune response of FPP003 were evaluated in a double-blind trial. Twenty healthy participants received either a low or high dose of FPP003 (*n* = 8, respectively) or placebo (*n* = 4) in three doses. Regarding safety, while mild to moderate adverse events were observed, no severe or serious adverse events occurred in all participants. For immunogenicity, the geometric mean fold rise (GMFR) in anti-IL-17A antibody was increased with peak at day 71 or 85 and continued until day 141. In ELISpot assay, the number of IL-4 and IFN-γ spots was increased in a dose dependent manner. Furthermore, circulating follicular helper T cells was significantly increased in the FPP003 high-dose group. The adjuvant-free FPP003 peptide vaccine could be well-tolerated and beneficial approach in human.

## Highlights

• Peptide vaccine FPP003 is composed of B cell epitope for IL-17A and T cell epitope (AJP001)
• No severe AEs, and FPP003 peptide vaccine was tolerated in a phase 1 clinical trial
• The anti-IL-17A antibody peaked at day 71 or 85 and persisted until day 141
• Seroconversion (≥4-fold antibody titer increase) occurred in 14/15 (93.3%) participants

## Graphical abstract

**Figure:**
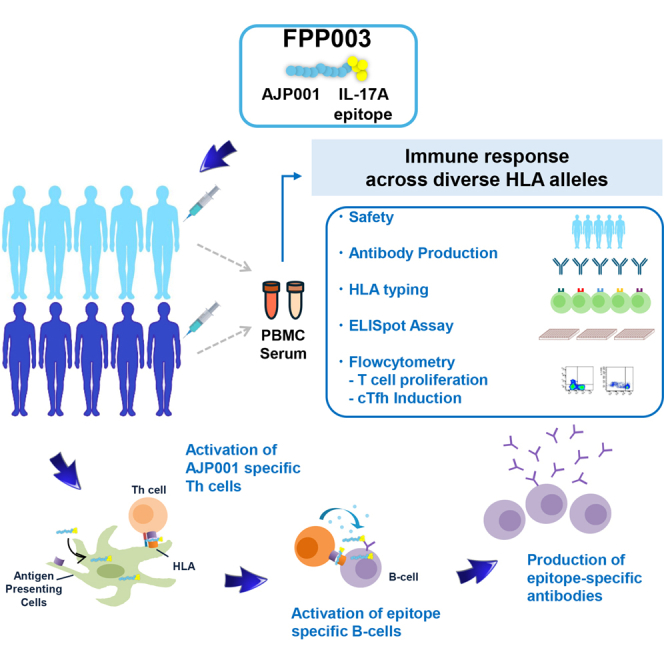

## Teaser

Immunology; Dermatology; Human genetics; Molecular medicine

## Introduction

Vaccine technology is now expanding its application to chronic diseases, such as Alzheimer’s disease, hypertension, and chronic inflammatory diseases (e.g., rheumatoid arthritis, psoriasis).^1^^,^^2^ These vaccines aim to suppress disease progression by utilizing the immune response to inhibit the function of target molecules, leading to improving patients’ quality of life. Antibody therapeutics that neutralize disease-related proteins, such as inflammatory cytokines or immune modulators, are currently widely used due to their high efficacy with continued growth.^3^^,^^4^^,^^5^ However, high costs of therapies have been pointed out as a significant burden on healthcare economics.^6^^,^^7^

To overcome these high-cost challenges and pursue sustained therapeutic effects, novel strategies such as therapeutic vaccines—including peptides, nucleic acids, and viral vectors—are gaining attention.^8^^,^^9^^,^^10^ Among these, peptide vaccines can be manufactured at low cost due to the simplicity of quality control through chemical synthesis.^11^ We challenge to resolve two critical problems: the technical and safety issues associated with adjuvant dependency, and the cost challenges of existing antibody therapeutics. Our technique precisely optimizes the arrangement and structure of specific B cell epitope and T cell epitope (AJP001) which also possesses innate immune activity,^12^ and enable the long-term production of antibodies against therapeutic targets without the use of any adjuvants.

In this study, we confirmed the safety and immune response of FPP003 when administered to healthy Japanese subjects. Exploratory analyses using peripheral blood mononuclear cells (PBMCs) were conducted to evaluate the induction of antigen-specific immunity in T cells, specifically the production of IL-4 and IFN-γ, and the induction of circulating follicular helper T cells (cTfh). Furthermore, we examined the correlation between the predicted binding affinity of FPP003 to HLA-DRB1 alleles and the induced antibody titers.

## Results

**Figure 1:**
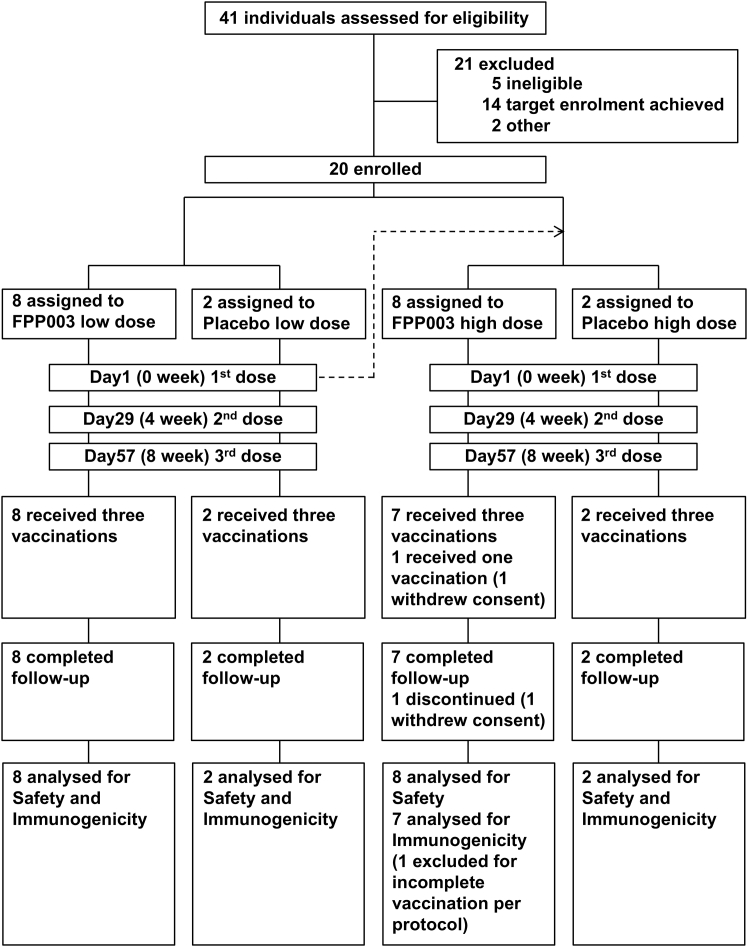
Study flow chart Participants were enrolled according to a dose-escalation protocol and randomly assigned within each dose cohort to receive FPP003 or placebo. Twenty participants were enrolled from forty-one participants and received three doses of either the FPP003 low-dose group (*n* = 8), placebo low-dose group (*n* = 2), FPP003 high-dose group (*n* = 8), or placebo high-dose group (*n* = 2) at 28 days interval. One participant did not receive the second and third vaccination: because of consent withdrawal (FPP003 high dose group). All participants completed 141 days of follow-up except the discontinued participant.

Twenty participants were enrolled from forty-one participants from April 22 to May 24, 2022 and received three doses of either the FPP003 low-dose group (*n* = 8), placebo low-dose group (*n* = 2), FPP003 high-dose group (*n* = 8), or placebo high-dose group (*n* = 2) at 28 days interval (Figure 1). The study enrolled an equal number of male and female participants (Table 1). One participant in the FPP003 high-dose group withdrew consent and discontinued the study after the first vaccination. All other participants completed all required visits and procedures, and the final follow-up for the last participant was completed on Oct 15, 2022. The trial concluded upon completion of this scheduled follow-up period, having achieved the target sample size with no additional discontinuations. Safety assessments were conducted in 20 subjects (including 8 in the high-dose group), while efficacy assessments were conducted in 19 subjects (including 7 in the high-dose group.

**Table 1:** Baseline characteristics

	Low dose	High dose	Placebo	Total
Number	8	8	4	20
Sex female; *n* (%)	4 (50%)	4 (50%)	2 (50%)	10 (50%)
Age (years); mean ± SD	43.6 ± 15.2	47.5 ± 13.2	47.3 ± 6.7	45.9 ± 12.7
Height (cm); mean ± SD	171.4 ± 10.6	165.9 ± 8.6	170.0 ± 12.0	168.9 ± 9.9
Weight (kg); mean ± SD	64.0 ± 9.1	66.0 ± 13.8	62.6 ± 15.8	64.5 ± 11.9
BMI (kg/m^2^); mean ± SD	21.7 ± 1.5	23.9 ± 4.1	21.3 ± 2.6	22.5 ± 3.1
Current smokers; *n* (%)	1 (12.5%)	2 (25%)	1 (25%)	4 (20%)

**Figure 2:**
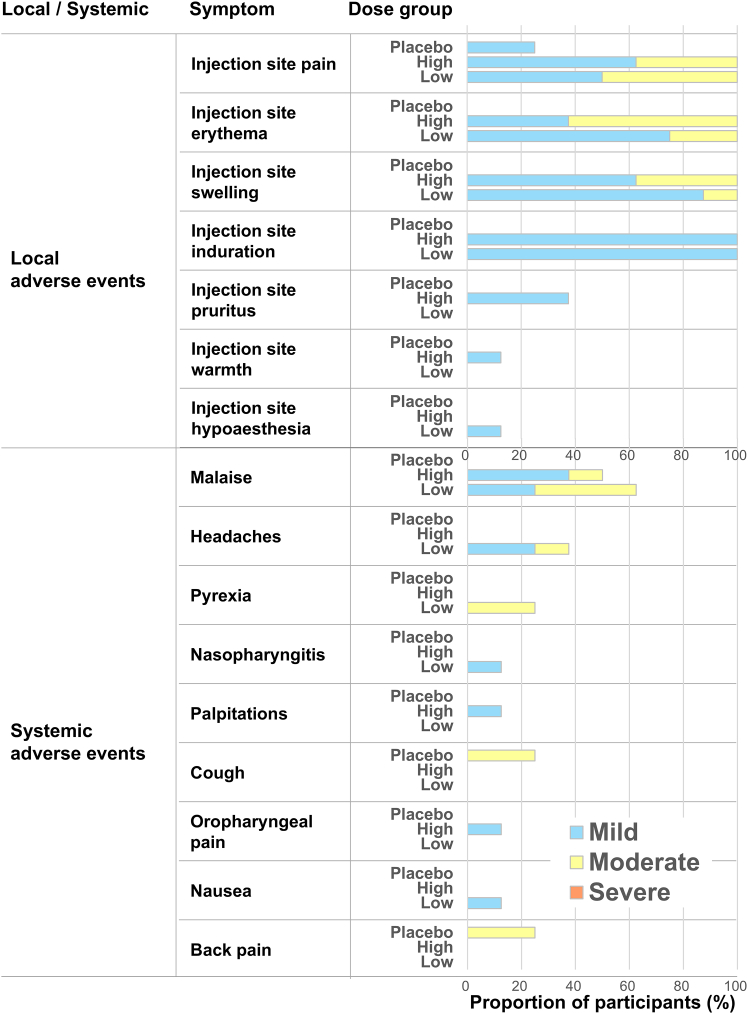
Frequency of participants with systemic and local adverse events and severity Proportion (percent) of participants was displayed separately as local and systemic adverse events for the placebo group (*n* = 4), the FPP003 high-dose group (*n* = 8), FPP003 low-dose group (*n* = 8). Severity was assessed for AEs according to toxicity grading scales modified and abridged from the US FDA guidance, shown as mild (blue bar), moderate (yellow bar), and severe (red bar). No grade 3 severe AEs were observed.

A summary of adverse events (AEs) was shown in Table 2. No severe AEs, serious AEs, or AEs leading to discontinuation were observed in either dose group in this study. AEs occurred in 100.0% of patients in the FPP003 low-dose and high-dose groups, respectively, and in the placebo group, AEs occurred in 50.0%. The main AEs were injection site reactions, with injection site pain, injection site erythema, injection site induration, and injection site swelling occurring at a high frequency of 100.0% in both the low and high FPP003 dose groups. In the placebo group, injection site pain occurred in 25.0% (Figure 2). The main systemic AEs were fatigue, which occurred frequently in 62.5% of participants in the low-dose FPP003 group and 50.0% in the high-dose group. Headache and fever occurred in 37.5% and 25.0% of participants in the low-dose FPP003 group but none in the high-dose group. Cough and back pain occurred in 25.0% of participants in the placebo group. Slightly abnormal laboratory values were scattered but were not considered AEs. There were no problematic abnormalities in vital signs.

**Table 2:** Summary of adverse events

	FPP003		
	Low dose	High dose	Placebo
Number	8	8	4
Adverse event (AE)	8 (100%)	8 (100%)	2 (50.0%)
Mild	1 (12.5%)	1 (12.5%)	0 (0.0%)
Moderate	7 (87.5%)	7 (87.5%)	2 (50.0%)
Severe	0 (0.0%)	0 (0.0%)	0 (0.0%)
related AE	8 (100%)	8 (100%)	1 (12.5%)
AE leading to discontinuation	0 (0.0%)	0 (0.0%)	0 (0.0%)
Serious AE	0 (0.0%)	0 (0.0%)	0 (0.0%)
Death	0 (0.0%)	0 (0.0%)	0 (0.0%)

**Figure 3:**
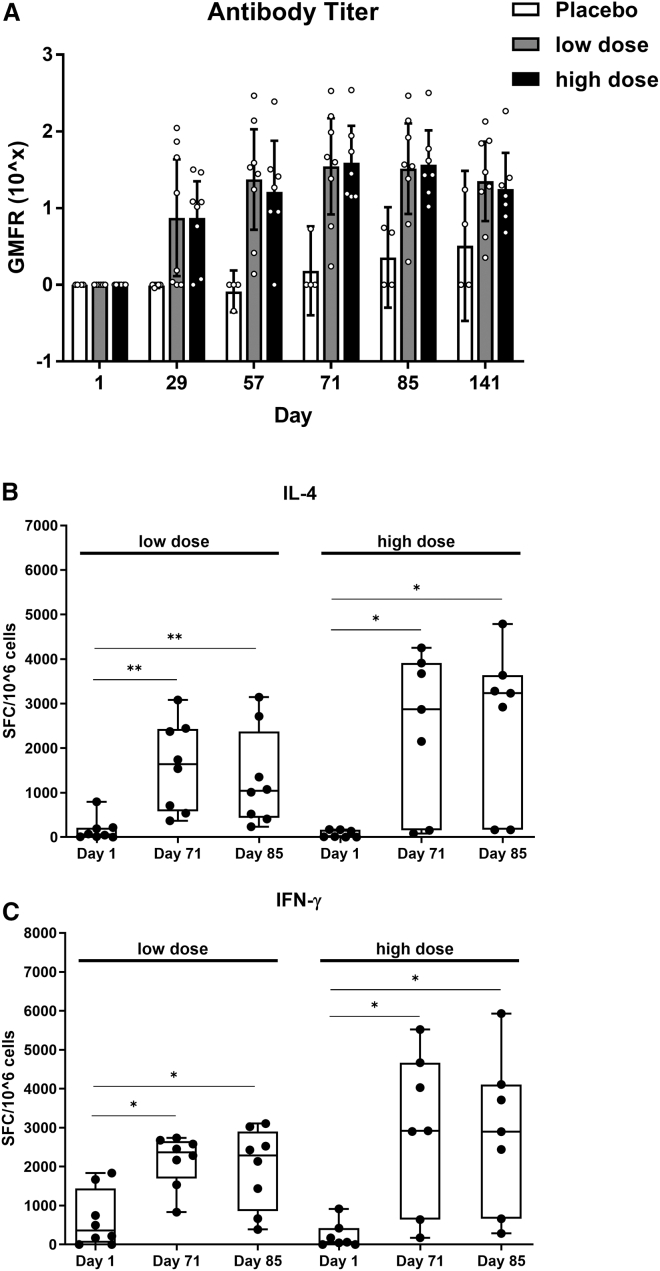
FPP003 induces anti-IL-17A antibody production and specific T cell response (A)The geometric mean fold-rise (GMFR) of anti-IL-17A antibody titer were shown on day 1 (before administration), 29, 57, 71, 85, and141 for the placebo group (*n* = 4), the FPP003 low-dose group (15 mg/body; *n* = 8), FPP003 high-dose group (24.8 mg/body; *n* = 7). Results are expressed as the geometric mean values ±95% confidence intervals (CIs). Dots present individual fold increase in anti-IL-17A antibody titer. (B and C) ELISpot assay, T cell response to FPP003, was measured on day 1 (pre-dose), day 71 and 85, for IL-4 (B) and IFN-γ (C). ELISpot assay and expressed as the cytokine spot-forming cells (SFC) per million PBMCs for the FPP003 high-dose group (*n* = 7), FPP003 low-dose group (*n* = 8). Results are expressed as median, interquartile range (IQR), and range (minimum and maximum). ∗*p* < 0.05, ∗∗*p* < 0.01 analyzed by Wilcoxon matched-pairs signed-rank test vs. day1.

For immunogenicity evaluation, anti-IL-17A antibody titers were measured on day 1 (before administration), 29, 57, 71, 85, and 141. The geometric mean fold rise (GMFR) in anti-IL-17A antibody titer for each treatment group is shown in Figure 3A. GMFR in the low and high FPP003 dose groups increased over time starting on day 29, with maxima of 35.08 (95% confidence interval [CI], 8.28–148.7) and 39.27 (95% CI, 3.02–118.4) at day 71, 32.67 (95% CI, 8.42–126.8), and 37.03 (95% CI, 3.03–103.1) at day 85 and continued until day 141, the last day of observation. GMFR trends were similar in the low and high FPP003 dose groups. GMFR in the placebo group fluctuated in the range of 0.82–3.22, with no significant increase. In the evaluation of gender differences in anti-IL-17A antibody titers, the GMFR was 22.18 (95% CI, 5.483–89.69) and 57.85 (95% CI, 19.05–175.7) in women. Although women tended to have higher values, no significant difference was observed.

In addition, ELISpot (Enzyme-Linked Immunospot) assay was utilized for T cell analysis. PBMCs were collected from participants receiving FPP003 or placebo on day 1 (pre-dose), 71, and 85. The number of IL-4 spots was significantly increased on day 71, 85 in the low and high FPP003 dose groups compared to day 1 (before treatment), whereas it was not changed in the placebo group (Figure 3B). Similarly, IFN-γ spot counts showed significant increases only in the low and high FPP003 dose groups at days 71 and 85 compared to day 1 (pre-dose) (Figure 3C). Both IL-4-positive and IFN-γ-positive cell counts showed an approximately 2-fold higher increase in the number of spots in the high-dose group compared to the FPP003 low-dose group. Furthermore, FPP003-specific T lymphocyte proliferative responses were evaluated by carboxyfluorescein succinimidyl ester (CFSE) dilution (Figure S1). T lymphocyte was increased on day 71 in both dose and on day 85 in the high FPP003 dose groups compared to day 1 (before treatment).

**Figure 4:**
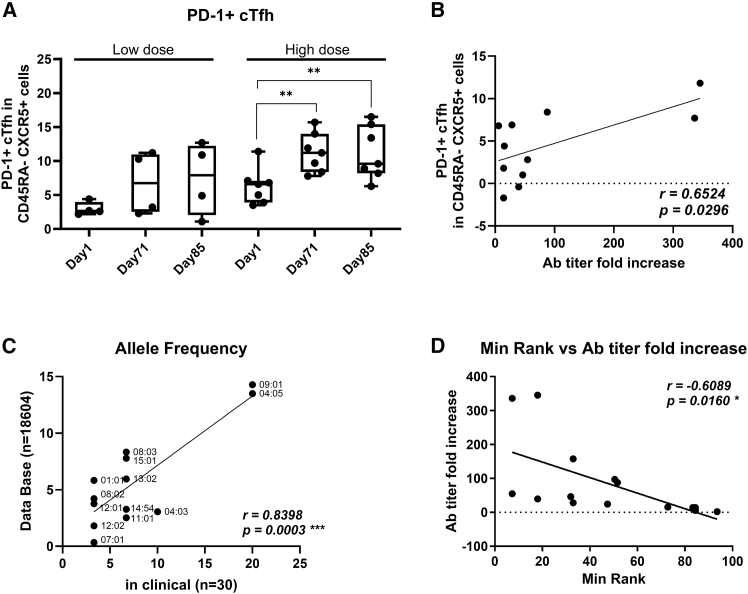
Correlation of FPP003-specific circulating follicular helper T cells and antibody titer (A) Increase of activated circulating follicular helper T cells (cTfh) was shown on day 1 (V2), 71(V6), and 85(V7) for the FPP003 high-dose (*n* = 7) and low-dose (*n* = 4) group, as indicated by the percentage of PD-1-positive cTfh cells in CD45RA^−^ and CXCR5^+^ cells. Results are expressed as median, interquartile range (IQR), and range (minimum and maximum). ∗∗*p* < 0.01 analyzed by Wilcoxon matched-pairs signed-rank test vs. V2. (B) Scatterplot showing the correlation between the increase in anti-IL-17A antibody titer (Ab Titer fold increase) and the induction of PD-1 positive cTfh in CD45RA^−^ and CXCR5^+^ cells on day 71 (*n* = 11). Statistical analysis was done by Pearson correlation coefficient (r = 0.6524, *p* = 0.0296). (C) The HLA-DRB1 of the FPP-treated participants was shown. Allele frequency was calculated in both FPP003-treated participants and the general Japanese population dataset (Japan pop 16 [*n* = 18,604], Allele Frequency Net Database). Statistical analysis was done by Pearson correlation coefficient (r = 0.8398, *p* = 0.0003). (D) Correlation between predicted affinity of FPP003 for HLA-DRB1 (*in silico*) and fold increase in anti-IL-17A antibody titer on day 71(*n* = 15). Min Rank is a value expressed as a %rank (percentile rank) of the predicted affinity. A lower %rank indicates a higher predicted affinity, and the Min Rank is derived from the allele with the higher predicted affinity out of the two existing alleles. Statistical analysis was done by Pearson correlation coefficient (r = −0.6089, *p* = 0.0160).

The activation of follicular T cells is important for the sustained increase of antibody titer.^13^ Therefore, we performed cTfh induction analysis by expanding FPP003-specific cTfh by adding FPP003 and IL-2 to PBMCs from subjects who received FPP003. Antigen-specific cTfh was identified PD-1^+^, ICOS^+^, and PD-1^+^ICOS^+^ cells in CD3^+^ CD4^+^ CD45RA^−^ CXCR5^+^ T cells (Figure S2). The percentage of PD-1-positive cTfh cells significantly increased in the FPP003 high-dose group on day 71(V6) and 85(V7) (Figure 4A). We further analyzed the data separately for CD4 and CD8 cells. Proliferated T cells were identified CFSE low cells in CD3^+^, CD4^+^, and CD8^+^ T cells (Figure S3). While there was a significant increase in CD4 cells at high doses, no increase was observed in CD8 cells (Figure S4). To analyze the immunogenicity of FPP003, we further evaluated whether there was a correlation between the increase in anti-IL-17A antibody titer and the induction of cTfh after FPP003 administration: on day 71, when the GMFR of anti-IL-17A antibody titer peaked after FPP003 administration, the fold increase in anti-IL-17A antibody titer was significantly correlated with the percentage of PD-1 positive cTfh cells (r = 0.6524, *p* = 0.0296) (Figure 4B).

The HLA-DLB1 of participants treated with FPP003 was examined and indicated in Figure 4C and Table S1. To evaluate the distribution of HLA-DRB1 allele frequencies in the subjects in this study, we compared them with those reported in an existing Japanese population database (Japan pop 16 (*n* = 18604), Allele Frequency Net Database, http://www.allelefrequencies.net/). A positive high correlation was observed in the scatterplot of both allele frequencies (Figure 4C). It indicates that the HLA-DRB1 allele frequency of the subject population in this study is well representative of the HLA-DRB1 allele frequency of the Japanese population. To examine the potential impact of the genetic background of the HLA-DRB1 allele on the immunogenicity of FPP003, we analyzed the relationship between the predicted affinity of FPP003 for each HLA-DRB1 allele in the study subjects and the fold increase in antibody titer measured on day 71 after FPP003 administration. Prediction of the affinity of FPP003 for each HLA-DRB1 allele was performed by NetMHCII 4.1 using the peptide sequence of AJP001, a helper T cell epitope of FPP003. The results showed that the lower the Min Rank value (higher predicted binding affinity) of AJP001 for a particular HLA-DRB1 allele, the higher the magnitude of increase in antibody titer and the more significant the correlation (r = −0.6089, *p* = 0.0160) (Figure 4D).

## Discussion

This study demonstrated that antibody titers could be induced in healthy subjects using a peptide vaccine without adjuvants. Peptide vaccine usually requires potent adjuvants, which not only complicate and increase the cost of manufacturing but also, particularly with long-term repeated administration, enhance the risk of systemic side effects and injection site reactions due to the adjuvant’s own non-specific immune-stimulating effects.^14^^,^^15^ Of importance, AJP001^12^ incorporated into the peptide sequence of FPP003 functions as a helper T cell epitope, inducing an immune response against self-proteins. Additionally, it exhibits adjuvant-like activity based on its innate immune activation capacity via TLR agonist-like effects. This combination enables immune response induction without adjuvants. Consequently, it maximizes the key advantages of peptide vaccines: safety, manufacturing simplicity, and low cost.

Regarding safety, although injection site reactions were observed at a high frequency, their severity was all mild (grade 1) to moderate (grade 2), suggesting a local immune response. The main systemic AEs were fatigue (56.3%), headache (18.8%), and fever (12.5%). A meta-analysis indicated that vaccines with AS01/AS02 and AS03 adjuvants exhibit higher reactogenicity, and the maximum incidence rates of systemic AEs were fever 61.8% and 22.6%, respectively; myalgia 56.7% and 21.3%; fatigue 46.3% and 30.5%; and headache 42.2% and 41.8%.^16^ Furthermore, adjuvant-containing vaccines suggest a relative risk of grade 3 fatigue, headache, and myalgia approximately 2.5–3 times higher than the control group. In contrast, in this study, no grade 3 systemic AEs were observed, and the overall frequency remained low. We evaluated the initial immune response following administration of FPP003 by measuring humans plasma concentrations of IL-6 and TNF-α by ELISA (R&D Systems) and white blood cell (WBC) count and C-reactive protein (CRP) levels; however, no changes in the systemic inflammatory markers were observed under the conditions of this study (data not shown). These results suggest that the adjuvant-free design of FPP003 may have suppressed the induction of systemic inflammation, thereby achieved the desired antibody induction while maintained tolerability.

Tfh are an essential cell population supporting long-term high-affinity antibody production in lymphoid tissues.^17^ Recently, we elucidated the factors that determine the longevity of spike-specific antibodies of SARS-CoV2 mRNA vaccines and traced the characteristics of spike-specific T cell clonotypes together with their epitopes and anti-S antibody titers before and after vaccination over time. Indeed, after the vaccination, T cell clonotypes highly responsive to recall spike stimulation were polarized to Tfh cells in donors exhibiting sustained anti-spike antibody titers.^18^ In our immunological analysis using PBMCs, the IL-4 and IFN-γ production were significantly increased, and cTfh was also induced that suggests the effective and sustained antibody response *in vivo*. Of importance, at day 71, when antibody titers peaked after investigational drug administration, a significant positive correlation (correlation coefficient r = 0.6524, *p* < 0.0296) was confirmed between the increase in anti-IL-17A antibody titers and cTfh induction (Figure 4B). These clinical data demonstrate that FPP003 efficiently activates germinal center (GC) responses without adjuvants, sustainably induces antibodies, and supplies Tfh cells necessary for B cell maturation and long-term memory formation.

This peptide vaccine using the T-epitope (AJP001) is HLA restricted. Indeed, this is consistent with findings showing a positive correlation between the *in silico* predicted binding affinity of FPP003 to HLA-DRB1 alleles and induced antibody titers (Figure 4D). As shown in Table S1, when vaccine responders were defined as subjects who showed a 4-fold or greater increase in antibody titers, 14 of the 15 subjects in the immunogenicity analysis population were vaccine responders (93.3%), and one was a non-responder (6.7%). The single non-responder was homozygous for the HLA-DRB1 allele with the lowest *in silico* predicted binding affinity, i.e., the highest %rank (DRB1∗04:05:01). To further analyze the relationship between alleles and the immunogenicity of this agent, we combined the alleles from cases showing increased antibody titers with their predicted binding affinities for FPP003. The major HLA-DRB1 alleles are DRB1∗09:01 (14.3%), DRB1∗04:05 (13.5%), DRB1∗15:02 (10.7%), DRB1∗08:03 (8.3%), DRB1∗15:01 (7.8%), and DRB1∗13:02 (6.0%). Indeed, the study population in this clinical trial also possessed these high-frequency HLA-DRB1 alleles, with the exception of DRB1∗15:02, and a strong positive correlation was observed in a scatterplot comparing the allele frequencies of the Japanese population database and the study population (Figure 4C). We then identified the HLA-DRB1 alleles present in each responder case as the responder allele, excluding the non-responder allele (DRB1∗04:05:01). The combined frequency of these identified responder alleles in the Japanese population was 61.1%. Based on this cumulative frequency and considering diploid genotype frequencies, the estimated response coverage rate for FPP003 was 84.9%. This coverage rate represents a high level for a response to a single antigen and enables coverage of most of the HLA diversity in the Japanese population. This result indicates that this drug can induce an effective immune response across a broad Japanese population. Furthermore, alleles predicted to have high binding affinity by *in silico* analysis do exist in reality as “potential responder alleles”, suggesting that the actual response coverage rate of FPP003 could potentially be even higher.

### Limitations of the study

We evaluated the neutralizing activity of vaccine-induced anti-IL-17A antibodies with purified IgG from serum samples collected on days 71 and 85 using a protein G column. As a result, no neutralizing activity was detected in this assay system required a purification process. In previously conducted nonclinical studies (rhesus monkeys), concentration-dependent neutralizing activity was confirmed with purified IgG from serum samples using a column coated with a fragment of recombinant human IL-17A. We speculate that the difference in sample purification methods had an impact. The aforementioned are the limitations of this study.

Overall, the adjuvant-free FPP003 peptide vaccine could be a helpful tool to address challenges related to the cost and safety of chronic disease treatment. The FPP003-induced immune response mediated by cTfh induction may support the mechanism of sustained antibody production and achieve broad HLA coverage. Future clinical trials are anticipated to determine whether it can similarly elicit a high and sustained immune response in actual chronic inflammatory diseases and to evaluate the therapeutic efficacy of this long-term immune response.

## Resource availability

### Lead contact

Further information and requests for resources and reagent should be directed to and will be fulfilled by the lead contact, Hironori Nakagami (nakagami.hironori.med@osaka-u.ac.jp).

### Materials availability

This study did not generate new unique reagents.

### Data and code availability

This study has been registered with jRCT: jRCT2051220007, and the full protocol is available as a supplementary appendix. We have made the dataset available once approval is obtained from the developer company.

This study does not report any original code.

Any additional information required to reanalyze the data reported in this paper is available from the lead contact upon reasonable request, subject to approval by the developer company.

## Acknowledgments

This work was supported by a grant from the 10.13039/100009619Japan Agency for Medical Research and Development, Japan,　Practical Research Project for Rare/Intractable Diseases (23ek0109565h0003). The investigational drug was supplied by FunPep Co., Ltd. We thank all the members of the Department of Health Development and Medicine for supporting this project.

## Author contributions

S.K., R.M., H.N., and T.T. designed and conducted the research; S.K., A.T., M.S., S.T., H.T., and H.N. acquired the data; S.T. and T.T. analyzed the data; and S.K., A.T., R.M., H.H., and T.T. wrote and edited the manuscript.

## Declaration of interests

The Department of Health Development and Medicine is an endowed department supported by the AnGes, Daicel, and FunPep. The Department of Clinical Gene Therapy is an endowed department supported by Novartis, AnGes, Shionogi, Boeringher, Fancl, Saisei Mirai Clinics, ROHTO, and FunPep. S.K., A.T., M.S., S.T., and H.T. are employed by FunPep. R.M. and H.N. are stockholders and scientific advisers for FunPep.

## STAR★Methods

### Key resources table

**Table:** 

REAGENT or RESOURCE	SOURCE	IDENTIFIER
**Antibodies**		

Anti-human IgG antibody (goat anti-human IgG H&L (HRP) preabsorbed	Abcam	Cat# Ab97175; RRID: AB_10680841
Human TruStain FcX™(Fc Receptor Blocking Solution), BioLegend, 422302	BioLegend	Cat# 422302; RRID:AB_2818986
Brilliant Violet 510™ anti-human CD3 Antibody	BioLegend	Cat# 317332; RRID:AB_2561943
APC/Cyanine7 anti-human CD4 Antibody	BioLegend	Cat# 344616; RRID:AB_2028483
FITC anti-human CD45RA	BioLegend	Cat# 304106; RRID:AB_314410
PE/Cyanine7 anti-human CD185 (CXCR5) Antibody	BioLegend	Cat# 356924; RRID:AB_2562355
PerCP-Cy™5.5 Mouse Anti-Human CD278	BD Pharmingen	Cat# 562833; RRID:AB_2737825
APC anti-human CD279 (PD-1) Antibody	BioLegend	Cat# 621610, RRID:AB_2832830

**Biological samples**		

Human serum	OPHAC Hospital	N/A
Human Peripheral Blood Mononuclear Cells (PBMC)	OPHAC Hospital	N/A

**Chemicals, peptides, and recombinant proteins**		

BSA conjugated peptide	PEPTIDE INSTITUTE, INC.	N/A
Carbonate bi-carbonate Coating Buffer pH 9.6	Medicago AB	Cat# 09-8922-100
PBS (tablet)	Merck KGaA	Cat# P4417
D-PBS (−)	FUJIFILM Wako	Cat# 045-29795
Goat serum	Kohjin Bio Co., Ltd.	Cat# 12180810
Tween 20	Merck KGaA	Cat# P7949
Skim Milk Powder	FUJIFILM Wako Pure Chemical Corporation	Cat# 190-12865
TMB substrate solution	SeraCare Life Science Inc.	Cat# 5120-0053
0.5 mol/L Sulfuric acid	Sigma-Aldrich Co. LLC.	Cat# 285940-5
ZOMBIE Violet Fixable Viability Kit	BioLegend	Cat# 423113
Cell Trace CFSE Cell Proliferation Kit	Thermo Fisher Scientific	Cat# C34554
Anti-Aggregate Wash (20×)	CTL	Cat# CTL-AA-010
Human AB Serum	MP Biomedicals	Cat# 2931949
FBS	SIGMA	Cat# 173012
Recombinant Human IL-2	Peprotech	Cat# 200-02

**Critical commercial assays**		

Human IFN-γ/IL-4 Double-Color Enzymatic ELISPOT Assay	CTL	Cat# hIFNgIL4-2M/10

**Software and algorithms**		

SoftMax Pro version 5.4	Molecular Devices	https://www.moleculardevices.com/products/microplate-readers/acquisition-and-analysis-software/softmax-pro-software; RRID:SCR_014240
Prism version 6.07	Graphpad	https://www.graphpad.com/features; RRID:SCR_002798
Immunospot 7.0.30.4	CTL	https://immunospot.com/immunospot-software.html; RRID:SCR_011082
BD FACSCanto II Flow Cytometer	BD Biosciences	BD FACSCanto™ Clinical Flow Cytometry System; RRID:SCR_018056
BD FACSDiva Software 8.0.1	BD Biosciences	https://www.bdbiosciences.com/en-us/products/software/instrument-software/bd-facsdiva-software; RRID:SCR_001456
NetMHCIIpan - 4.1	DTU Health Tech	https://services.healthtech.dtu.dk/services/NetMHCIIpan-4.1/
Allele Frequency Net Database	Allele Frequency Net Database	https://www.allelefrequencies.net/default.asp; RRID:SCR_007259
SAS (Ver. 9.4)	SAS Institute Inc.	https://www.sas.com
Microsoft Excel 365	Microsoft Corporation	https://www.microsoft.com

### Experimental model and study particpant details

#### Study design

This was a single-center, randomized, double-blind, placebo-controlled phase 1 study in a dose-escalation manner consisting of two cohorts to evaluate safety, tolerability and immunogenicity. This study was conducted at a specialized clinical research hospital in Osaka, Japan. Healthy subjects were randomized to receive FPP003 or a placebo in a 4:1 ratio within each cohort. The random allocation sequence was performed using a computer-generated program. The FPP003 low-dose 15 mg cohort (*n* = 10) was initiated first, and safety was evaluated one week after the first dose in all subjects in this cohort. Following confirmation by the investigator that there were no safety issues, the high-dose 24 mg cohort (*n* = 10) was initiated. The investigational product was administered three times at 4-week intervals.

The high-dose cohort was not initiated if either of the following criteria was met in the low-dose cohort: 1. serious adverse event occurred in at least one patient, and the causal relationship to the study drug was judged to be “definite,” “probable,” or “possible.” 2. A non-serious adverse event with high severity occurs, and the causal relationship to the study drug was judged to be “definite,” or “probable.” Additionally, the event remained unresolved in at least one patient one week after the first administration.

#### Blinding

An independent investigational site pharmacist, who was not otherwise involved in the study, managed the allocation codes and prepared/dispensed the investigational products. Although the active drug and placebo vials had distinguishable appearance, the pharmacist prepared and drew the solutions into identical syringes in a restricted area. Consequently, the final dispensed study drugs were identical in appearance, maintaining complete blinding for participants, investigators, and all other study personnel.

#### Trial oversight

The study was approved by the Institutional Review Board of Medical Corporation Heishinkai OPHAC Hospital (Ref. No. 1129 PB), conducted according to the principles of the Declaration of Helsinki, and was registered on jRCT: jRCT2051220007. All patients provided written informed consent before enrollment.

#### Investigational medicinal product and placebo

FPP003 is a peptide vaccine composed of 28 natural amino acid residues, containing 20 amino acid helper T cell epitope (AJP001), 8 amino acid human IL-17A-derived B-cell epitope, and an aminocaproic acid linker connecting the two epitopes. The amino acid sequences from 65aa to 72aa were selected as B-cell epitope for IL-17A.

The investigational product used in this study was a subcutaneous injection, provided in 1 mL vials with a concentration of 30 mg/mL of FPP003. Acetate buffer was used as the placebo, which did not contain FPP003.

#### Study population

Male and female participants between 20 and 65 years, and healthy with no clinically significant abnormalities as determined by medical history, physical examination, 12-lead electrocardiogram (ECG), and clinical laboratory evaluations, were eligible for inclusion. All participants were required to have a negative PCR test for SARS-CoV-2, or negative results for both SARS-CoV-2 IgM and SARS-CoV-2 IgG antibodies, or to have received at least two doses of a COVID-19 vaccine.

Females were required to be postmenopausal, defined as having had a final menstrual period at least 12 months prior to informed consent. Male participants whose sexual partners could become pregnant were required to use medically acceptable method of contraception from the time of informed consent until 12 weeks after the first dose of the investigational product.

Key exclusion criteria included: a history of inflammatory bowel disease; active or latent tuberculosis infection, a suspicion of tuberculosis infection, or a history of tuberculosis; receipt of a COVID-19 vaccine, live or inactivated vaccine, or a toxoid within 4 weeks of study start, or a plan to receive one between the start of the study and Day 85; receipt of any medication, such as immunomodulators, immunosuppressants, or biologics, that could affect the immune system within 4 weeks of study start; and positive test results for HIV, hepatitis B, or hepatitis C during the screening phase.

#### Trial procedures and outcomes

The investigational product was administered subcutaneously in the central deltoid region three times at 4-week intervals. Participants in the FPP003 low-dose cohort received a single 0.5 mL injection, which was administered to either the left or right arm. Participants in the FPP003 high-dose cohort received two 0.4 mL injections, one in each arm. After each injection, participants were kept at rest and observed at the study site for at least 30 min to monitor for any reactions.

The primary endpoints of this study were safety and immunogenicity. Safety was evaluated by collecting information on the type, frequency, and severity of adverse events (AEs) and serious AEs. AEs were collected from the time of first injection through the end of the observation period (Day 141) and were graded as mild, moderate, or severe according to the protocol-specified criteria. The immunogenicity of the study drug was assessed by analyzing the geometric mean fold-rise (GMFR) of anti-IL-17A antibody titer during the observation period.

HLA genotypes for seven loci (HLA-A/B/C, DPB1, DRB1, DQA1, and DQB1) were determined prior to the administration of the investigational product. As an exploratory evaluation, the relationship between these HLA genotypes and anti-IL-17A antibody titer was analyzed. Additionally, the relationship between the HLA genotypes and antigen-specific immune responses, including ELISpot assay (IFN-γ, IL-4), cTfh (circulating follicular helper T) induction, and T cell proliferation, was also analyzed.

### Method details

#### Serum and PBMC isolation and cryopreservation

To determine anti-IL-17A antibody titer in serum, blood sample was collected at 1 (pre-dose), 29, 57, 85 and 141 days after first dosing. Blood was collected into BD Vacutainer SST Tubes (BD) and mixed gently by inverting the tube five-six times. Tube was allowed to clot by leaving them undisturbed at room temperature for 30 min, then centrifuged at 1100–1300×*g* for 10 min at 4 °C. Supernatant (serum) was subdivided into polypropylene tube (SARSTEDT) and stored in medical freezer setting at −85°C until shipment to test facility.

Whole blood sample was collected at 1 (pre-dose), 71 and 85 after first dosing to evaluate antigen specific immune response. Whole blood was collected into BD Vacutainer CPT Tubes with Sodium Heparin (BD) and immediately inverting tubes 8–10 times gently to mix blood and anticoagulant and avoid micro clotting. Tube was centrifuged at 1500–1800×*g* for 15 min at room temperature in a horizontal rotor (swing-out head) within 2 h after blood collection. After centrifuge, tube was inverted 5–10 times gently and transferred to test facility under condition from 18°C to 25°C. In test facility, cell suspension above the gel was collected with a Pipette and transferred to 50 mL centrifuge tube. D-PBS (−) (FUJIFILM Wako) was added into tube at 30 mL, mixed gently and centrifuge at 300×*g* for 15 min at room temperature. Cell pellet was resuspended and washed with D-PBS (−) twice and cell number was counted. The concentration of PBMC was adjusted to 10^6^–10^7^ cells/mL in CELL BANKER 1(Zenyaku Kogyo Company, Limited, Cat. CB013), transferred into cryotube (Thermo SCIENTIFIC) and placed in pre-chilled Mr. Frosty Freezing Container (Thermo Scientific) in medical freezer more than 24 h. Frozen Cryotube was transferred to Nitrogen vapor tanks and stored until immune response assay.

#### Antibody titer assay

Anti-IL-17A antibody titer was determined using ELISA. BSA conjugated peptide of human IL-17A-derived B-cell epitope (PEPTIDE INSTITUTE, INC.) dissolved in carbonate buffer (pH 9.6) was added at 10 μg/mL to Immuno Plate (Thermo Fisher Scientific) and incubated for 2 h at 25°C. Solution in plate was discharged, added goat serum (Kohjin Bio Co., Ltd.) for blocking and incubated for 2 h at 25°C. After blocking, serum sample diluted at 10 times in goat serum was serially diluted with a common ration of 3, added to each well of the plate and incubated for 22 h under refrigeration. The plate was washed with PBS (Merck KGaA) containing 0.05% Tween 20 and then anti-human IgG antibody (goat anti-human IgG H&L (HRP) preabsorbed, Abcam) was added and incubated for 3 h at 25°C. The plate was washed, added TMB substrate solution (SeraCare Life Science Inc.) and incubated for 30 min protected from light. 0.5 mol/L Sulfuric acid (Sigma-Aldrich Co. LLC.) was added to stop reaction, and absorbance (measurement wavelength: 450 nm, reference wavelength: 650 nm) was measured using microplate reader (VERSAmax, Molecular Devices Japan K.K.) within 5 min.

Regression curve (x-axial: Dilution factor, y-axial: absorbance 450 nm–650 nm) was present using 4-parameter formula by SoftMax Pro (Molecular Devices Japan K.K., ver.5.4). From the regression curve obtained from sample, a dilution factor was calculated with the absorbance of 1.5 and defined as the antibody titer.

#### IL-4 and IFN-γ ELISpot assay

FPP003 specific IL-4 and IFN-γ secreting T cells induced by FPP003 administration were determined by ELISpot assay. Cryopreserved PBMCs were thawed and suspended in RPMI1640 (Thermo Fischer Scientific) supplemented with 5% human AB serum (MP Biomedicals) and 2-mercaptoethanol (Thermo Fischer Scientific) at the cell density of 2–3 × 10^6^ cells/mL and seeded in 96 well round bottom plate. For FPP003-specific clonal expansion, PBMCs were stimulated with 2 μg/mL of FPP003 for about 10 days and recombinant human IL-2 was added every 2–4 days at the final concentration of 1 ng/mL.^19^^,^^20^ Expanded PBMCs were collected and analyzed by ELISpot assay (Human IFN-γ/IL-4 Double-Color Enzymatic ELISPOT Assay, CTL). In brief, expanded PBMCs were added in the 96-well ELISPOT plates coated with human IFN-γ/IL-4 capture antibody at 1–1.5 × 10^5^ cells/100 μL/well and stimulated by FPP003 at the concentration of 2 μg/mL. Culture medium was added for the negative control well and PMA/Ionomycin was served as positive control at 100 ng/mL. After 48 h incubation, spots were detected with human IFN-γ/IL-4 Detection Antibody, Tertiary Solution, Blue Develop Solution and Red Develop Solution as following manufacturer’s instruction. Spots were counted using CTL-ImmunoSpot S6 Core Analyzer. The intensity of T cell responses to FPP003 was calculated by spot forming unit per million cells normalized by respective negative control.

#### TFH induction

FPP003 specific circulating Tfh (cTfh) induction was analyzed by flow cytometry. In brief, FPP003 specific T cells were expanded as same protocol as described in IL-4 and IFN-γ ELISpot assay. After the culture with IL-2, PBMCs were collected, and Fc receptor was blocked by Human TruStain FcX (Fc Receptor Blocking Solution). Cells were stained in staining buffer using ZOMBIE Violet, BV510 anti-human CD3, APC-Cy7 anti-human CD4, FITC anti-human CD45RA, PE/Cyanine7 anti-human CD185 (CXCR5), PerCP-Cy5.5 anti-Human CD278 (ICOS), APC anti-human CD279 (PD-1). Stained cells were washed twice and resuspended in staining buffer. All samples were analyzed by FACS Canto II cytometer (BD) and evaluated by FACS Diva Software (BD).

#### T lymphocyte proliferative responses

FPP003 specific T cell proliferation was evaluated by carboxyfluorescein succinimidyl ester (CFSE) dilution. Cryopreserved PBMCs were thawed, washed with PBS(−) and stained by CFSE. Cells were suspended in 0.2 μM CFSE in PBS(−) and incubated for 5 min at 37°C. The staining was stopped by adding complete cell culture media. After washing, cells were resuspended in the medium and cultured overnight with or without the treatment of FPP003 (2 μg/mL). Culture medium was added for the control well and PMA/Ionomycin was served as positive control at 100 ng/mL.

#### HLA genotypes

HLA genotypes were measured at centralized lab (Hokenkagaku Nishinihon).

### Quantification and statistical analysis

The sample size was determined as an appropriate number of initial evaluation subjects to assess the safety and tolerability of the investigational product. Stratified block randomization was applied according to sex within each dose cohort, using a fixed block size of 5 (4 active and 1 placebo per block). For summary statistics on safety and immunogenicity (antibody titers), we used the following: For continuous variables, the number of subjects (n), arithmetic mean, standard deviation (SD), minimum, Q1, median, Q3, and maximum were used. For continuous immunogenicity variables, the geometric mean and its 95% confidence interval, along with the arithmetic mean and standard deviation of the log-transformed values, were used instead of the arithmetic mean and standard deviation. Categorical variables were summarized using the number and percentage of subjects.

The number of IL-4 and IFN-γ-producing cells (measured by ELISpot assay) and the proportion of PD-1-positive cells among cTfh cells were analyzed using the Wilcoxon signed-rank test. Pearson’s correlation coefficient was used to analyze the relationship between the fold increase in antibody titer, the proportion of PD-1-positive cells in cTfh cells, and allele frequency. Statistical analysis and chart creation were performed using SAS (Ver. 9.4), GraphPad Prism (version 6.07), and Microsoft Excel 365.

### Additional resources

This study has been registered with jRCT: jRCT2051220007, and the full protocol is available as a supplementary appendix.
