# Supplementary material for: Safety and immunogenicity of an adjuvant-free peptide vaccine targeting IL-17A: Phase 1 randomized controlled trial
Source: iScience. 2026 Jul 21;29(8):116817. doi: 10.1016/j.isci.2026.116817 (PMC13393739; doi:10.1016/j.isci.2026.116817)
Supplement: Document S1. Figures S1–S4 and Table S1 [file mmc1.pdf]

iScience, Volume *29*

Supplemental information

Safety and immunogenicity of an adjuvant-free peptide vaccine targeting IL-17A: Phase 1 randomized controlled trial

Satoru Kasahara, Akiko Tenma, Makoto Sakaguchi, Shigeyoshi Tsuji, Hideki Tomioka, Ryuichi Morishita, Hironori Nakagami, and Tetsuya Tomita

Supplement Figure 1

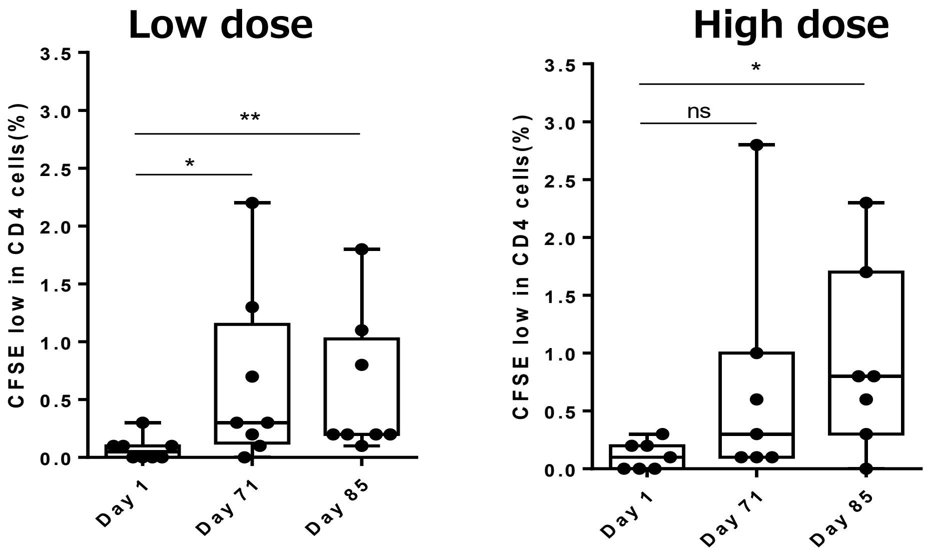

Supplement Figure 1 FPP003-specific CD4 T cell proliferative responses
CD4 T cell proliferation was assessed by carboxyfluorescein succinimidyl ester (CFSE) dilution , with the percentage of proliferating CD4 cells (defined as CFSE-low) shown on Day 1 (pre-dose), Day 71 and 85 for the FPP003 low-dose group (n=8), FPP003 high-dose group (n=7). Results are expressed as median, interquartile range (IQR), and range (minimum and maximum).
*:p<0.05, **:p<0.01 analyzed by Wilcoxon matched-pairs signed-rank test vs. Day1.

Supplement Figure 2

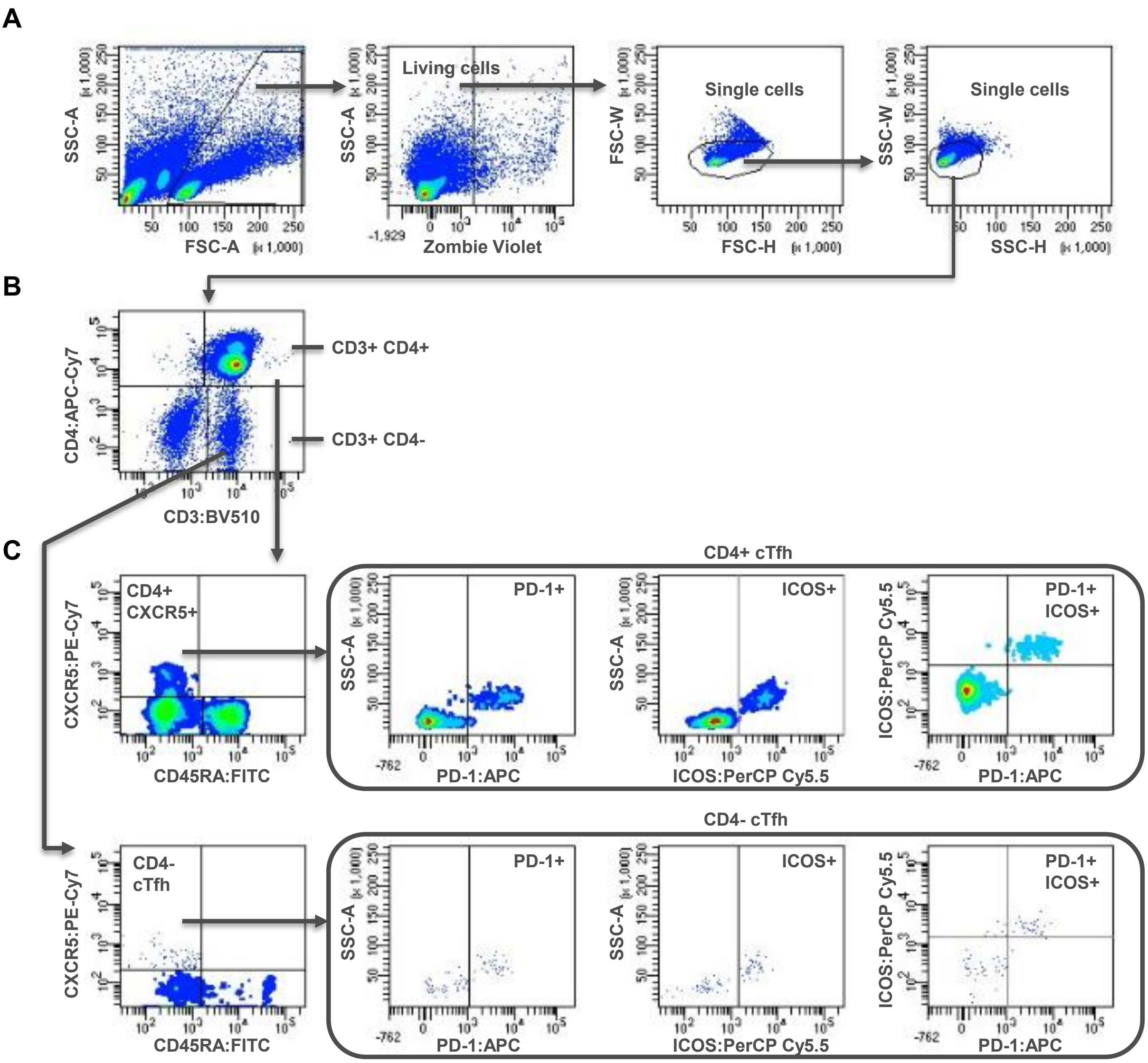

Supplement Figure 2. Gating Strategy for antigen-specific cTfh cells
(A) Gating strategy to identify live lymphocytes in PBMC. Lymphocytes were identified by forward scatter area (FSC-A) and side-scatter area (SSC-A). Dead cells were excluded with Zombie violet staining and doublets were excluded by gating on single cells as determined by FSC-H versus FSC-W and SSC-H versus SSC-W. (B) CD3/CD4 dot-plot was used for CD3+CD4+ and CD3+CD4- T cells gating. (C) CD45RA/CXCR5 dot-plots were used to identify cTfh in CD4+ and CD4- T cells and subsequent plots show the representative gating for PD-1+, ICOS+ and PD-1+ICOS+ cells within cTfh cells.

Supplement Figure 3

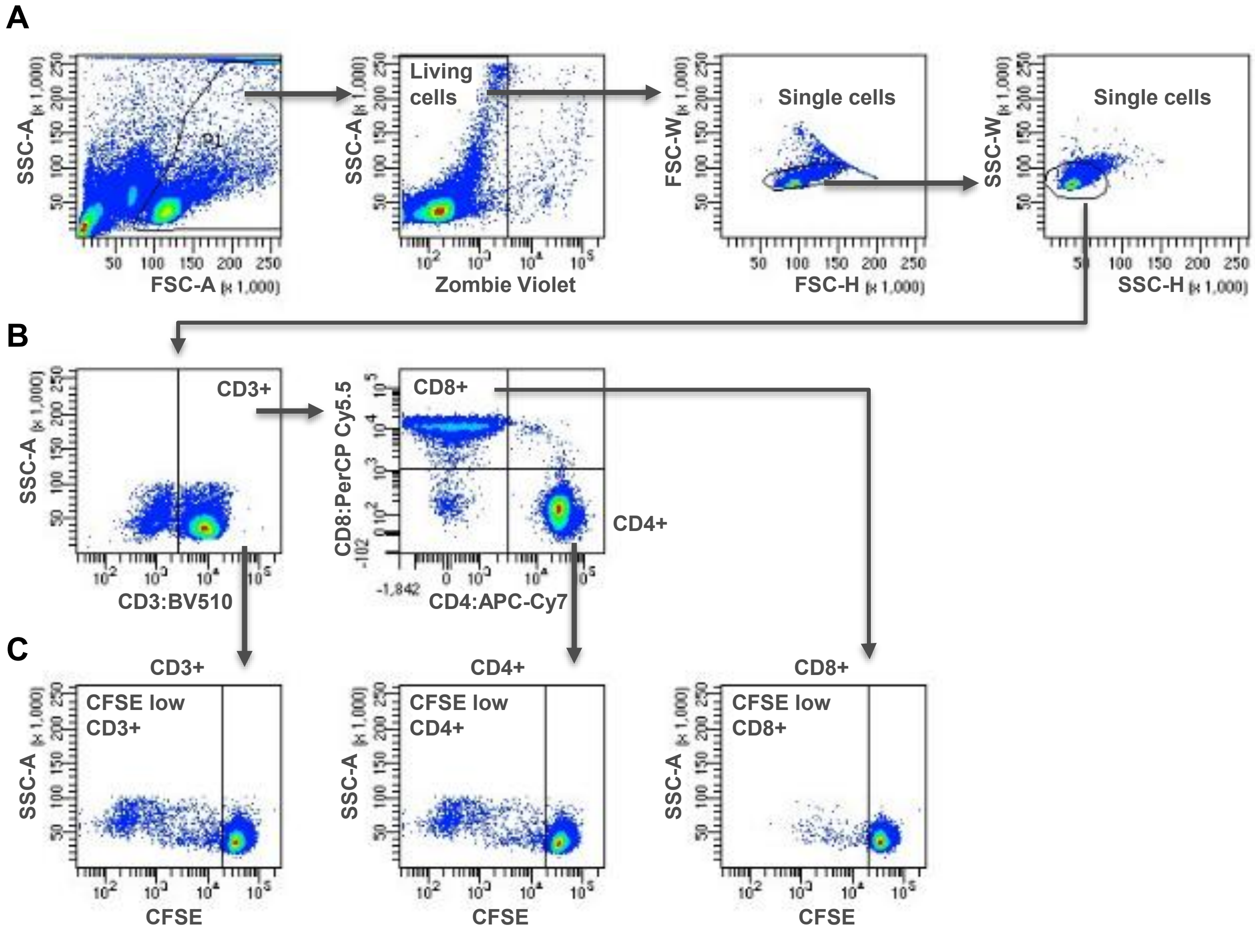

Supplement Figure 3. Gating Strategy for Proliferated T cells.
(A) Gating strategy to identify live lymphocytes in PBMC. Lymphocytes were identified by forward scatter area (FSC-A) and side-scatter area (SSC-A). Dead cells were excluded with Zombie violet staining and doublets were excluded by gating on single cells as determined by FSC-H versus FSC-W and SSC-H versus SSC-W. (B) CD3 and CD4/CD8 dot-plot was used for CD3+CD4+ and CD3+CD8+ T cells gating. (C) Proliferated T cells in CD3+, CD4+ and CD8+ T cells were identified by CFSE dilution.

Supplement Figure 4

A

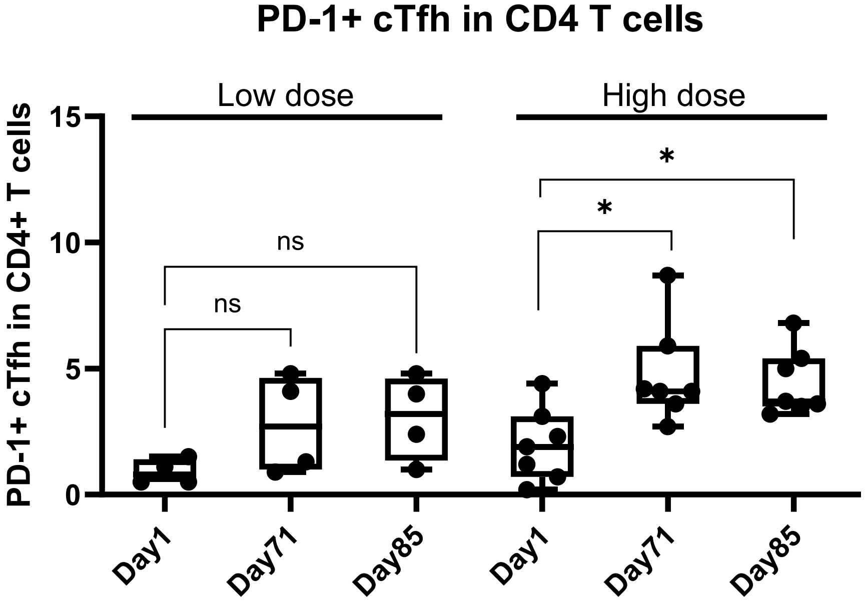

B

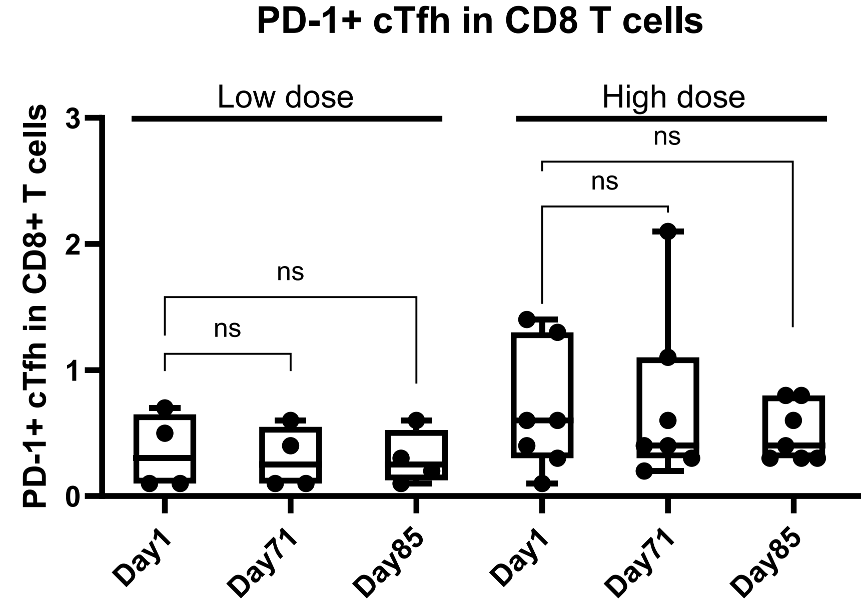

Supplement Figure 4. Induction of activated circulating follicular helper T cells. Population of activated circulating follicular helper T cells were shown on Day 1 , Day 71 and 85 for the FPP003 high-dose (n=7) and low-dose (n=4) group, as indicated by the percentage of PD-1-positive cTfh cells (CD45RA- and CXCR5+) in (A) CD4+ and (B) CD8+ T cells. Results are expressed as median, interquartile range (IQR), and range (minimum and maximum).

*:p<0.05 analyzed by Wilcoxon matched-pairs signed-rank test vs. Day 1

Supplement Table 1

Subject ID	HLA-DRB1		% Rank*			Fold increase in antibody titer**
	allele 1	allele 2	allele 1	allele 2	Min Rank	
1	9:01:02	11:01:01	84.37	17.99	17.99	345.2
2	4:03:01	13:02:01	84.81	7.27	7.27	336.0
3	9:01:02	15:01:01	84.37	32.91	32.91	157.8
4	14:54:01		50.44		50.44	97.0
5	4:03:01	8:03:02	84.81	51.58	51.58	87.8
6	4:05:01	13:02:01	93.49	7.27	7.27	54.6
7	9:01:02	12:01:01	84.37	31.9	31.9	46.3
8	11:01:01	12:02:01	17.99	34.79	17.99	39.6
9	8:03:02	15:01:01	51.58	32.91	32.91	28.2
10	4:05:01	8:02:01	93.49	47.37	47.37	24.2
11	1:01:01	9:01:02	72.75	84.37	72.75	15.4
12	4:05:01	7:01:01	93.49	83.25	83.25	14.2
13	4:03:01	9:01:02	84.81	84.37	84.37	14.1
14	4:05:01	9:01:02	93.49	84.37	84.37	5.8
15	4:05:01		93.49		93.49	1.7

Supplement Table 1 HLA-DRB1 alleles, predicted affinity of FPP003 for each allele, and fold increase in anti-IL-17A antibody titer from FPP003-treated subjects.
Min Rank is a value expressed as a %rank (percentile rank) of the predicted affinity. A lower %rank indicates a higher predicted affinity, and the Min Rank is derived from the allele with the higher predicted affinity out of the two existing alleles.
* Predicted by NetMHCII 4.1 (NetMHCIIpan 4.1 - DTU Health Tech - Bioinformatic Services)
**Fold increase in anti-IL-17A antibody titer on Day 71 relative to the Day 1 baseline.
